# Genetic Susceptibility Loci, Pesticide Exposure and Prostate Cancer Risk

**DOI:** 10.1371/journal.pone.0058195

**Published:** 2013-04-04

**Authors:** Stella Koutros, Sonja I. Berndt, Kathryn Hughes Barry, Gabriella Andreotti, Jane A. Hoppin, Dale P. Sandler, Meredith Yeager, Laurie A. Burdett, Jeffrey Yuenger, Michael C. R. Alavanja, Laura E. Beane Freeman

**Affiliations:** 1 Division of Cancer Epidemiology and Genetics, National Cancer Institute, National Institutes of Health, Department of Health and Human Services, Bethesda, Maryland, United States of America; 2 Epidemiology Branch, National Institute of Environmental Health Sciences, National Institutes of Health, Department of Health and Human Services, Research Triangle Park, North Carolina, United States of America; 3 Core Genotyping Facility, Advance Technology Program, National Cancer Institute – Frederick, Frederick, Maryland, United States of America; Baylor College of Medicine, United States of America

## Abstract

Uncovering SNP (single nucleotide polymorphisms)-environment interactions can generate new hypotheses about the function of poorly characterized genetic variants and environmental factors, like pesticides. We evaluated SNP-environment interactions between 30 confirmed prostate cancer susceptibility loci and 45 pesticides and prostate cancer risk in 776 cases and 1,444 controls in the Agricultural Health Study. We used unconditional logistic regression to estimate odds ratios (ORs) and 95% confidence intervals (CIs). Multiplicative SNP-pesticide interactions were calculated using a likelihood ratio test. After correction for multiple tests using the False Discovery Rate method, two interactions remained noteworthy. Among men carrying two T alleles at rs2710647 in EH domain binding protein 1 (*EHBP1*) SNP, the risk of prostate cancer in those with high malathion use was 3.43 times those with no use (95% CI: 1.44–8.15) (P-interaction  = 0.003). Among men carrying two A alleles at rs7679673 in *TET2*, the risk of prostate cancer associated with high aldrin use was 3.67 times those with no use (95% CI: 1.43, 9.41) (P-interaction  = 0.006). In contrast, associations were null for other genotypes. Although additional studies are needed and the exact mechanisms are unknown, this study suggests known genetic susceptibility loci may modify the risk between pesticide use and prostate cancer.

## Introduction

Occupational exposure to pesticides has been associated with increased prostate cancer risk in many epidemiologic studies [1]–[6]. Specifically, several groups or chemicals classes have been linked to prostate cancer, including triazine herbicides [1], [7], [8], organochlorine insecticides (OC) [8]–[11], and organophosphate insecticides (OP) [8], [12], [13], but none of the associations are conclusive and it is unclear which specific pesticides might be driving the group findings. In the Agricultural Health Study (AHS), a prospective cohort of licensed private and commercial pesticide applicators from Iowa and North Carolina, we have consistently observed an excess of prostate cancer among AHS men compared to men in the general populations of Iowa and North Carolina [14], [15] and with continued follow-up of this cohort we recently reported an excess risk of prostate cancer associated with four insecticides, fonofos (OP), terbufos (OP), malathion (OP), and aldrin (OC) [16].

Genome-wide association studies (GWAS) have identified several independent single nucleotide polymorphisms (SNPs) as risk factors for prostate cancer [17]–[27]. Since these discoveries, large epidemiologic studies have also attempted to uncover SNP-environment interaction in the hopes of generating new hypotheses about the function of many of these gene poor regions. Similarly, SNP-environment interactions help inform our understanding of potential mechanisms by which an environmental factor might influence risk. We previously reported interactions between the organophosphate (OP) insecticide fonofos and known susceptibility loci in the 8q24 region and significant increased risks of prostate cancer, suggesting that variants identified from GWAS may interact with environmental factors [28]. With increasing information about the function of the 8q24 region in cancer development [29]–[31], this finding provides valuable information about how pesticide use might act to influence prostate cancer risk.

In this study, we use newly genotyped data in 32 prostate GWAS SNPs to continue to explore possible SNP-pesticide interactions and risk of prostate cancer in 2,220 AHS subjects included in a nested case-control study.

## Materials and Methods

### Study population

The AHS is a prospective cohort study that includes 55,747 male licensed pesticide applicators in Iowa and North Carolina, recruited from 1993 through 1997 [32]. During a follow-up interview conducted in 1999–2003, applicators were asked for a mouthwash rinse sample to provide DNA from buccal cells. Approximately 72% of all applicators who completed the follow-up interview returned a buccal sample. In addition, applicators with incident prostate cancer who had not returned a sample at follow-up were asked separately to provide one, with 307/561 (55%) returning a sample. White male pesticide applicators diagnosed with incident prostate cancer between 1993 and 2004 were included in the current nested case-control study. Eligibility, inclusion and exclusion criteria have been previously described [28]. Briefly, cancer cases were coded using the *International Classification of Diseases for Oncology*, 2^nd^ edition, and stage (local, regional, distant, unstaged) and grade (well differentiated, moderately differentiated, poorly differentiated, undifferentiated, missing) were abstracted by the state cancer registries in Iowa and North Carolina. Eligible controls were frequency matched 2∶1 to cases by date of birth (+/− 1 year). Controls were white, male applicators who provided buccal cell material, were alive and not lost to follow-up at the time of case diagnosis, and had no previous cancer diagnosis except non-melanoma skin cancer. Based on these inclusion criteria, 841 cases (66% of total white cases in the cohort as of 2004) and 1,659 controls were identified (total N = 2,500). Due to genotyping space limitations 164 controls were excluded. Of the remaining samples, 108 were removed due to insufficient or poor DNA quality (N = 20; 14 cases, 6 controls) or <90% completion rate (i.e. more than 10% of the SNP assays failed for a given sample, N = 88; 47 cases, 41 controls). We further identified 5 individuals who were suspected to be non-white (<80% European ancestry using STRUCTURE software [33] or significant deviation from the first two components in principal components analysis [34]) leaving a final sample size of 776 cases and 1,444 controls. Participants provided written informed consent, and the study protocol was approved by the institutional review boards of the National Institutes of Health, the University of Iowa, and other contractors in compliance with all applicable requirements of the United States.

### Genotyping and Quality Control

Thirty-two SNPs not previously genotyped in the AHS but reported as susceptibility loci from GWAS of prostate cancer [17]–[27] were evaluated. Genotyping was performed at NCI's Core Genotyping Facility (http://cgf.nci.nih.gov/operations/uniplex-genotyping.html) [35], using Applied Biosystems TaqMan® SNP Genotyping Assays. SNPs with low completion rate (<90% of samples) were excluded (rs1465618 and rs4962416). The mean genotyping rate was 96% for the remaining 30 SNPs. No SNPs showed evidence of deviation from Hardy-Weinberg proportions given alpha = 0.05/30 = 0.0017 after Bonferroni correction. Blinded duplicate samples (5%) were also included and concordance of these samples was 100%.

### Statistical Analysis

Unconditional logistic regression was used to estimate odds ratios (ORs) and 95% confidence intervals (CIs) for the association between SNPs and prostate cancer and the interaction between SNPs and pesticide use with prostate cancer risk. For SNP associations, genotypes were coded as counts of the risk allele assuming a log-additive model and models were adjusted for age (10 yr-intervals) and state (Iowa or North Carolina). Information on lifetime use of 50 pesticides was captured in two self-administered questionnaires completed during cohort enrollment. All nested case-control study participants completed the first (enrollment) questionnaire, which inquired about ever/never use of the 50 pesticides, as well as duration and frequency of use for a subset of 22 of the pesticides, while 1,439 of these men (60.4% of cases and 67.2% of controls), completed the second (take-home) questionnaire, which inquired about use of the remaining 28 pesticides. Pesticides with a prevalence of use less than 5% in the current nested case-control subgroup were excluded leaving 45 pesticides for analysis (17 herbicides, 21 insecticides, 2 fumigants, and 5 fungicides); a list of all 45 pesticides and their prevalence of use is presented in Table S1. Cumulative lifetime exposure to each pesticide was assessed by intensity-weighted lifetime exposure days and categorized into three groups (non-exposed, low, and high, with low and high divided at the median among controls for each pesticide). SNP-pesticide interactions, adjusted for age and state, were examined in a multiplicative model using the three-level pesticide variable and assuming the dominant genetic model for SNPs. The P-value (1 df) for each SNP-pesticide interaction was computed by comparing nested models with and without the cross-product terms using a likelihood ratio test. SNP-pesticide combinations with a P-interaction<0.05 and a significant increased risk (α = 0.05) of prostate cancer following a monotonic pattern with increasing pesticide exposure in one genotype group and no significant association in the other group are presented. We also evaluated pesticide interactions with a cumulative score variable (continuous and categorical) by coding genotypes as zero, one, or two risk alleles to assess the contribution of multiple independent SNPs (n = 26, including those from 8q24 [28]) and prostate cancer risk. All P-values are two-sided and all analyses were performed using AHS data release version P1REL0712.04.

We applied the false discovery rate (FDR) (Benjamini – Hochberg adjustment) method to account for the expected proportion of false discoveries. FDR values were calculated separately for each pesticide from the results of 30 tests (i.e., total number of SNPs evaluated) in the evaluation of the association between each SNP-pesticide interaction and the risk of prostate cancer. Interactions were deemed noteworthy at an FDR  = 0.20 level.

## Results

Applicators in the current study were representative of applicators in the larger cohort with respect to a variety of demographic characteristics [28]. Also, cases were similar in age, state of residence, and applicator type to controls in the study but had a higher proportion of first-degree relatives with a family history of prostate cancer compared with controls (16.7% versus 10.0%, Table 1).

**Table 1:** Selected characteristics of prostate nested case-control participants.

	Cases		Controls		
Selected Characteristics	n	%	n	%	Chi square p-value
**All subjects**	776	100.0	1,444	100.0	
**Age (years)**					
<40	12	1.5	17	1.2	
40–49	138	17.8	273	18.9	
50–59	369	47.6	673	46.6	
60–69	219	28.2	408	28.3	
≥70	38	4.9	73	5.1	0.91
	776				
**State of Residence**					
Iowa	520	67.0	991	68.6	
North Carolina	256	33.0	453	31.4	0.44
**Applicator Type**					
Private	741	95.5	1,363	94.4	
Commercial	35	4.5	81	5.6	0.27
**First-degree family history of prostate cancer**					
No	576	74.2	1,193	82.6	
Yes	130	16.8	145	10.0	<.0001
**Prostate Cancer Stage**					
I – Local	578	74.5	-	-	
II – Regional	156	20.1	-	-	
III – Distant	12	1.5	-	-	
IV – Not staged	30	3.9	-	-	-
**Prostate Cancer Grade**					
Well differentiated	38	4.9	-	-	
Moderately differentiated	547	70.5	-	-	
Poorly differentiated	168	21.6	-	-	
Undifferentiated	4	0.5	-	-	
Not graded	19	2.4	-	-	-

All observed associations for the 30 SNPs and prostate cancer were in the same direction as reported in GWAS of prostate cancer [17], [18], [22]–[27], [36], [37] (Table 2), except for rs12500426 (*PDLIM5*) for which the opposite allele was observed to be the risk allele compared to the initial report [17]. Among the 30 genotyped SNPs, the strongest association was with the *MSMB* SNP rs10993994 (p-trend = 0.0002, Table 2). Additionally, there were eight loci with 0.001<P-trend <0.01 (rs1859962, rs5759167, rs2710647, rs4430796, rs7841060, rs902774, rs17632542, rs16901979) and three loci with 0.01<P-trend <0.05 (rs10896449, rs266849, rs10486567).

**Table 2:** Risk of prostate cancer in the AHS for previously reported susceptibility loci identified from genome wide association studies of prostate cancer.

Region	SNP	Known gene/region	Risk Allele	RAF*	OR**95% CI	P-trend
2p15	rs721048	*EHBP1*	A	0.17	1.20 (1.00, 1.45)	0.051
2p15	rs2710647	*EHBP1*	C	0.53	1.24 (1.07, 1.43)	0.004
2p21	rs1465618†	*THADA*	–	–	–	–
2q31	rs12621278	*ITGA6*	A	0.94	1.06 (0.78, 1.43)	0.714
2q37	rs2292884	*MLPH*	G	0.23	1.17 (0.99, 1.37)	0.061
3p11	rs7629490	Intergenic	T	0.34	1.05 (0.90, 1.22)	0.560
3p12	rs2660753	Intergenic	T	0.10	1.05 (0.83, 1.32)	0.704
3q21	rs4857841	*EEFSEC*	A	0.28	1.13 (0.97, 1.31)	0.126
4q22	rs12500426	*PDLIM5*	C	0.56	1.09 (0.94, 1.25)	0.258
4q22	rs17021918	*PDLIM5*	C	0.64	1.03 (0.89, 1.18)	0.742
4q24	rs7679673	*TET2, PP2A*	C	0.62	1.10 (0.96, 1.28)	0.180
6q25	rs9364554	*SLC22A3*	T	0.60	1.11 (0.95, 1.29)	0.180
7p15	rs10486567	*JAZF1*	C	0.77	1.20 (1.01, 1.43)	0.036
7q21	rs6465657	*LMTK2*	C	0.45	1.13 (0.98, 1.31)	0.088
8q21	rs4961199	*CPNE3*	C	0.84	1.01 (0.84, 1.23)	0.909
8q24	rs16901979	Intergenic, HapC	T	0.03	1.60 (1.13, 2.28)	0.009
8q24	rs7841060	Intergenic, Region2	G	0.20	1.26 (1.06, 1.48)	0.007
8p21	rs1512268	*NKX3.1*	A	0.43	1.14 (0.99, 1.32)	0.069
8p21	rs13264338 surrogate for rs2928679	*SLC25A37*	C	0.44	1.10 (0.97, 1.27)	0.143
10q11	rs10993994	*MSMB*	T	0.31	1.30 (1.13, 1.50)	0.0002
10q26	rs4962416†	*CTBP2*	–	–	–	–
11q13	rs10896449	Intergenic	G	0.52	1.20 (1.04, 1.38)	0.012
11p15	rs7127900	*IGF2, IGF2AS, INS, TH*	T	0.19	1.10 (0.92, 1.31)	0.289
12q13	rs902774	*KRT8, EIF4B, TENC1*	T	0.14	1.29 (1.07, 1.56)	0.007
17q12	rs4430796	*HNF1B*	T	0.51	1.19 (1.06, 1.35)	0.005
17q24	rs1859962	Intergenic	G	0.46	1.25 (1.09, 1.45)	0.002
19q13	rs17632542	*KLK3*	T	0.91	1.38 (1.09, 1.74)	0.007
	rs266849		A	0.79	1.23 (1.05, 1.44)	0.013
	rs2735839		G	0.84	1.19 (1.00, 1.42)	0.051
22q13	rs5759167	*TTLL1, BIK*	C	0.49	1.26 (1.09, 1.45)	0.002
22q13	rs600173 surrogate for rs9623117	*TNRC6B*	T	0.77	1.04 (0.88, 1.23)	0.661
Xp11	rs5945619	*NUDT10, NUDT11*	G	0.37	1.12 (0.92, 1.38)	0.265

Single Nucleotide Polymorphism (SNP); Odds Ratio (OR); Confidence Interval (CI); Agricultural Health Study (AHS).

* Risk Allele Frequency (RAF) among controls. ** OR per risk allele assuming a log-additive model. Adjusted for age and state.

† Completion rate <90%.

rs600173-rs9623117 r^2^ = 1.0, rs13264338-rs2928679 r^2^ = 0.96.

Stratified odds ratios for the association between pesticide use and prostate cancer for interactions <0.05 and a significant increased risk of prostate cancer following a monotonic pattern are presented in Table 3. Among men carrying two T alleles at rs2710647 in EH domain binding protein 1 (*EHBP1*), the risk of prostate cancer in those with low malathion use was 2.17 times those with no use (95% CI: 0.91, 5.14) and in those with high malathion use was 3.43 times those with no use (95% CI: 1.44–8.15) (P-interaction  = 0.003). Among men carrying two A alleles at rs7679673 in *TET2*, the risk of prostate cancer associated with low aldrin use was 1.86 times those with no use (95% CI: 0.73, 4.75) and for high aldrin use was 3.67 times those with no use (95% CI: 1.43, 9.41) (P-interaction  = 0.006). In contrast, associations were null for other genotypes. After correction for multiple tests, both of these interactions remained noteworthy at the FDR = 0.20 level.

**Table 3:** Stratified odds ratios and 95% CI, adjusted for age and state, for associations between pesticides and prostate cancer.

			Pesticide Use						
			None		Low		High		
SNP/Region	Pesticide	Genotype	Ca/Co		Ca/Co	OR (95% CI)	Ca/Co	OR (95% CI)	P-interaction
*EHBP1*	MALATHION	TT	9/50	REF	24/65	2.17 (0.91, 5.14)	28/50	3.43 (1.44, 8.15)	0.003*
rs2710647		CT+CC	95/192	REF	99/211	0.96 (0.68, 1.36)	91/223	0.80 (0.56, 1.15)	
*TET2*	ALDRIN	AA	22/82	REF	10/21	1.86 (0.73, 4.75)	13/14	3.67 (1.43, 9.41)	0.006*
rs7679673		AC+CC	204/444	REF	39/111	0.79 (0.52, 1.20)	51/117	0.97 (0.67, 1.42)	
17q24	TERBUFOS	TT	65/194	REF	28/55	1.72 (0.98, 3.03)	28/47	2.05 (1.16, 3.64)	0.037
rs1859962		GT+GG	242/486	REF	78/146	1.06 (0.77, 3.03)	70/151	0.92 (0.66, 1.28)	
*PDLIM5*	TERBUFOS	CC	121/290	REF	48/85	1.38 (0.91, 2.11)	46/71	1.59 (1.03, 2.45)	0.042
rs17021918		CT+TT	185/392	REF	60/116	1.09 (0.75, 1.58)	53/129	0.87 (0.60, 1.26)	

* Noteworthy at an FDR  = 0.20 level.

Among men carrying the variant allele at the *PDLIM5* SNPs rs1859962 or rs17021918 increased prostate cancer risk was observed with high compared to no terbufos use (OR = 2.05, 95% CI: 1.16–3.64, P-interaction = 0.037), (OR = 1.59, 95% CI: 1.03–2.45, P-interaction = 0.042), respectively (Table 3). Although nominally significant without adjustment for multiple testing, these interactions were not noteworthy after adjustment using the FDR method.

No interactions were observed between cumulative genetic score and pesticide use in relation to prostate cancer risk (data not shown).

## Discussion

We observed four quantitative interactions between GWAS loci and select pesticide use and risk of prostate cancer. Two of these, malathion-rs2710647 and aldrin-rs7679673, were noteworthy at the FDR  = 0.20 level after correction for multiple testing. Additional interactions with terbufos were also observed with a lesser level of significance. Interestingly, all of the observed interactions are with pesticides that have been implicated in the AHS as risk factors for aggressive prostate cancer [16].

The interactions with the OP insecticides malathion and terbufos were in one nongenic region on chromosome 17q24 and two gene regions, *EHBP1* and *PDLIM5*. The function of the rs1859962 SNP, which is located in a nongenic region, is not known. Although the nearest protein-coding regions, *KCNJ2* and *SOX9*, are ∼1Mb away, *SOX9* is involved in prostate epithelial differentiation and observed to promote prostate tumor cell proliferation when upregulated [38], [39]. *EHBP1* encodes an Eps15 homology domain binding protein, which is involved in clathrin-mediated endocytosis, a process fundamental to neurotransmission, signal transduction and the regulation of many plasma membrane activities. Alterations (fusions, somatic mutations, over and under-expression) of clathrin-mediated endocytosis proteins have been reported in numerous cancers, including prostate cancer [40]. *PDLIM5* (PDZ and LIM domain 5, also called *ENH* or *ENH1*) is a PDZ-LIM protein. PDZ-LIM proteins can act as signal modulators, influence actin dynamics, regulate cell architecture, and control gene transcription [41]. Misregulated PDZ-LIM proteins have been shown to promote tumor cell invasion and metastasis in prostate tumors and prostate cancer cell lines [42], [43]. Interestingly, the OP pesticides malathion and terbufos are acetylcholinesterase (enzyme that degrades the neurotransmitter acetylcholine) inhibitors. PDLIM5 is observed to be expressed in various brain regions and is localized in presynaptic nerve terminals where neurotransmitter vesicles are stored [44]. Although it is not clear how pesticides may interact with these variants to increase the risk of prostate cancer, it is possible that exposure to these pesticides may alter important signal transduction pathways and/or compromise cellular morphology to promote the development of carcinogenesis.

Another interaction was observed for the organochlorine (OC) insecticide aldrin and SNP rs7679673 on chromosome 4. This SNP is located between two gene regions, *TET2*, a gene recently characterized as a tumor suppressor gene involved in the pathogenesis of several hematopoietic diseases [45], and *PP2A*, a gene implicated in androgen regulation in prostate cancer cell lines [46]. Organochlorine pesticides, like aldrin, have been implicated as endocrine disrupting chemicals and may alter androgen levels to influence prostate cancer risk [47]. Although there is no direct information about the function of rs7679673, this variant has been shown to be associated with earlier onset of disease and to have a stronger association with prostate cancer among those with a family history of prostate cancer [17], [48]. In the AHS, we observed a significant interaction between aldrin and family history of prostate cancer [16]. Small numbers in the current analysis preclude evaluation of the effect of family history on the aldrin-rs7679673-prostate cancer association (3-way interaction).

Although we observed interesting interactions, the sample size for the current study is limited. This limited sample size is reflected by the small cell counts for some gene-exposure groups and in the inability to achieve the same magnitude of effect observed in GWAS for all SNP associations. This does not negate the importance of these SNPs in our population because they are known risk variants for prostate cancer as established by GWAS. We also considered many interactions in the current analysis thus our findings may be due to chance, however, after adjusting for multiple comparisons some interactions stood out. When interpreting the meaningfulness of an interaction between two factors, more credence is given to a positive interaction when each component has been shown to be a risk factor for the disease independently. Thus, it is important to note that the interactions we found in our study were observed between well-established GWAS loci and use of two specific pesticides (malathion and aldrin) that have independently been associated with prostate cancer in the AHS [16].

For many gene-exposure studies, a key limitation is the quality of the exposure information. In the AHS, we have high quality information on lifetime use of specific pesticides from several detailed questionnaires, which has been shown to be reliable [49], [50]. Few studies have the ability to examine interactions between pesticide exposure and genetic risk factors for prostate cancer, thus replication of these findings may be difficult.

In conclusion, we observed several positive interactions between pesticide use and GWAS loci. Interactions between the chromosome 2p15 SNP rs2710647 and malathion, as well as the chromosome 4q24 SNP rs7679673 and aldrin, were noteworthy after correction for multiple testing. Although additional studies are needed and the exact mechanisms by which these variants may interact with these specific pesticides are unknown, our study raises some intriguing questions about interplay of genetic and environmental risk factors for prostate cancer.

## Supporting Information

Table S1 List of 45 chemicals evaluated for interaction.
(DOC)
