# Supplementary material for: Genetic Susceptibility Loci, Pesticide Exposure and Prostate Cancer Risk
Source: PLoS One. 2013 Apr 4;8(4):e58195. doi: 10.1371/journal.pone.0058195 (PMC3617165; doi:10.1371/journal.pone.0058195)
Supplement: Table S1 — List of 45 chemicals evaluated for interaction. (DOC) [file pone.0058195.s001.doc]

| **Pesticide** | **Type** | **Prevalence of use (% Ever)** | |
| --- | --- | --- | --- |
|  |  | **Cases n (%)** | **Control n (%)** |
| Atrazine | Herbicide | 567 (75) | 1056 (74) |
| Dicamba | Herbicide | 360 (53) | 757 (57) |
| Cyanazine | Herbicide | 304 (44) | 633 (48) |
| Chlorimuron-ethyl | Herbicide | 243 (33) | 415 (30) |
| Metolachlor | Herbicide | 326 (47) | 626 (47) |
| EPTC | Herbicide | 150 (22) | 250 (19) |
| Alachlor | Herbicide | 420 (60) | 806 (60) |
| Metribuzin | Herbicide | 295 (41) | 579 (42) |
| Paraquat | Herbicide | 135 (19) | 298 (22) |
| Petroleum Oil/Petroleum Distillate | Herbicide | 237 (33) | 395 (29) |
| Pendimethalin | Herbicide | 264 (36) | 521 (38) |
| Imazethapyr | Herbicide | 278 (40) | 542 (41) |
| Glyphosate | Herbicide | 573 (76) | 1098 (77) |
| 2,4,5 T P | Herbicide | 64 (9) | 141 (10) |
| Butylate | Herbicide | 227 (31) | 463 (34) |
| Trifluralin | Herbicide | 392 (56) | 749 (56) |
| 2,4-D | Herbicide | 617 (82) | 1208 (85) |
| 2,4,5 T | Herbicide | 229 (31) | 469 (34) |
| Permethrin | Insecticide | 139 (20) | 260 (20) |
| Terbufos | Insecticide | 291 (42) | 523 (39) |
| Fonofos | Insecticide | 183 (26) | 332 (25) |
| Lindane | Insecticide | 124 (17) | 276 (20) |
| Carbofuran | Insecticide | 261 (38) | 461 (35) |
| Chlorpyrifos | Insecticide | 306 (40) | 571 (40) |
| Malathion | Insecticide | 526 (70) | 999 (71) |
| Parathion | Insecticide | 102 (14) | 181 (13) |
| Carbaryl | Insecticide | 395 (53) | 767 (55) |
| Diazinon | Insecticide | 220 (30) | 409 (30) |
| Aldicarb | Insecticide | 56 (8) | 97 (7) |
| Phorate | Insecticide | 271 (37) | 521 (38) |
| Aldrin | Insecticide | 252 (34) | 465 (34) |
| Chlordane | Insecticide | 230 (31) | 486 (35) |
| Dieldrin | Insecticide | 66 (9) | 151 (11) |
| DDT | Insecticide | 375 (50) | 684 (49) |
| Heptachlor | Insecticide | 187 (26) | 353 (26) |
| Toxaphene | Insecticide | 144 (20) | 272 (20) |
| Coumaphos | Insecticide | 68 (10) | 138 (11) |
| DDVP | Insecticide | 87 (13) | 190 (14) |
| Methyl Bromide | Fumigant | 116 (15) | 212 (15) |
| Carbon Di/Tetrasulfide | Fumigant | 58 (8) | 124 (9) |
| Benomyl | Fungicide | 75 (10) | 133 (10) |
| Chlorothalonil | Fungicide | 46 (6) | 101 (7) |
| Captan | Fungicide | 71 (10) | 157 (12) |
| Maneb | Fungicide | 69 (9) | 133 (10) |
| Metalaxyl | Fungicide | 147 (20) | 264 (19) |
